# Treatment indications and potential off‐label use of antidepressants among older adults: A population‐based descriptive study in Denmark

**DOI:** 10.1002/gps.5841

**Published:** 2022-11-15

**Authors:** Kazi Ishtiak‐Ahmed, Xiaoqin Liu, Kaj Sparle Christensen, Christiane Gasse

**Affiliations:** ^1^ Department of Affective Disorders Aarhus University Hospital Psychiatry Aarhus Denmark; ^2^ Department of Clinical Medicine Aarhus University Aarhus Denmark; ^3^ NCRR‐The National Centre for Register‐based Research Aarhus University Aarhus Denmark; ^4^ Department of Public Health Aarhus University Aarhus Denmark; ^5^ Research Unit for General Practice Aarhus University Aarhus Denmark

**Keywords:** general practice, geriatric depression, off‐label use, prescription without treatment indication

## Abstract

**Objectives:**

Off‐label prescriptions of antidepressants may be of special concern in older‐adults. We aimed to study the potential off‐label use of antidepressants among adults ≥65 years by describing the patterns, trends, and factors associated with missing and unspecified treatment indications.

**Methods:**

We used registry data to describe indications of all antidepressant prescriptions (*N* = 13.8 million) redeemed by older‐adults in 2006–2019. We investigated factors associated with off‐label use by considering prescriptions with missing and unspecified indications of the first antidepressant prescription using a multinomial logistic regression with the ‘depression’ indication as a reference category and reported odds ratios (ORs) with 95% confidence intervals (CI).

**Results:**

Overall, 18.1% of all antidepressant prescriptions had missing indications, and 9.9% had unspecified indications. The proportion of potential off‐label use based on missing and unspecified prescriptions remained mostly consistent during 2006–2019. We identified similar associations in user characteristics whether considering missing or unspecified first prescription. ORs with 95% CI were raised in non‐western ethnicity (vs. Danish, 1.12 (0.99–1.26) for missing indication and 1.28 (1.11–1.48) for unspecified indication) and female sex (vs. male, 1.05 (1.02–1.07) and 1.05 (1.02–1.07) respectively). ORs were reduced for shorter educational attainment (vs. long, 0.90 (0.87–0.94) and 0.92 (0.88–0.96)), older age (≥81 vs. 67–70 years, 0.66 (0.65–0.71) and 0.73 (0.70–0.76)) and hospital psychiatric diagnosis (per diagnosis 0.76 (0.73–0.78) and 0.88 (0.86–0.91)).

**Conclusions:**

Nearly one‐third of all antidepressant prescriptions redeemed by older‐adults in Denmark had either missing or unspecified treatment indications. Whether these prescriptions were actual off‐label use needs to be validated. Clinicians should pay special attention to patients' characteristics linking missing and unspecified indications and maintain adequate documentation while prescribing medication.

## Key points

- Nearly one‐third of all antidepressant prescriptions utilized by older adults are likely to be potential off‐label use.
- Patients' characteristics, including age, sex, educational attainment, marital status, place of residence, and past psychiatric diagnoses, are linked to potential off‐label antidepressant use.
- A risk‐benefit assessment of potential off‐label use of antidepressants in older adults is urgent.

## 1 INTRODUCTION

Major depression (depression) is the most common mental disorder in the older population (≥65 years), with a prevalence of up to 10%.^1^ Antidepressants are the first‐line pharmacological treatment for depression and depressive symptoms in all ages. Antidepressants are also used for treating other mental disorders, including panic disorder, social phobia, generalized anxiety disorder, obsessive‐compulsive disorder (OCD), post‐traumatic stress disorder, and specific types of pain.^2^ Although the use of antidepressants is considered beneficial,^3^ over half of older adults do not respond to initial antidepressant treatment,^4^ and some may experience side effects.^5^ Besides, antidepressants also increase the risk of falls, hyponatremia, stroke, and other complications.^4^, ^5^, ^6^ Moreover, age‐related pharmacokinetics and pharmacodynamics might alter the benefits of antidepressant use.^7^

Although it is necessary to ensure older adults' access to antidepressant treatment, ensuring appropriate use is paramount.^8^ Globally, there has been a surge in the use of antidepressants in recent decades.^9^ The growing trend of unapproved/off‐label use of antidepressants could be a contributing factor, among others.^10^ Off‐label medication can be defined as using a specific medicine for a purpose or in a dosage not in compliance with the authorized product information. Off‐label use of antidepressant prescriptions in primary care has been reported as high as 50% in the general population in Canada.^11^ Like‐wise, in older adults, a recent German study reported that more than 40% of all antidepressant prescriptions in 2009–2015 were off‐label use.^12^ We are not aware of similar data from Denmark. However, using a proxy measure (discharge diagnosis of hospital in‐ and outpatient contacts), recent Danish studies reported a high level of off‐label use in the general population.^13^, ^14^

Nonetheless, in Denmark, although the use of antidepressants has gradually decreased in recent years in all age groups, including older adults,^15^ it remains among the countries with the highest use of antidepressants. Clinicians in Denmark, including general practitioners (GPs), can prescribe off‐label medications in the context when no other suitable option exists within the approved medications and with the obligation of patient caring and conscientiousness in mind.^16^ When prescribing a medication, the prescriber selects an indication code from a list of approved indications or writes free text if the indication is out of the list or opts to leave the indication as empty.^17^ The free text indications are yet to be ready for use in research and are coded as unspecified, and the empty indications are coded as missing indications. As a rough measure, one way to assess potential off‐label by indication could be studying these prescriptions with missing and unspecified treatment indications.

Besides clinical factors, patients' socio‐demographics play a critical role in the treatment because patient‐related factors influence access to care, awareness of health, and decision‐making capacity in the treatment process.^18^ Similarly, patient‐related factors may also influence the treatment indications for which one uses antidepressants, even though prescription indications should corroborate the patients' clinical manifestation of the illnesses. Identifying and acknowledging patient‐related factors concerning treatment indication of antidepressants are essential to point the direction for future interventions targeting potential sub‐groups in older adults with over‐or underusing of antidepressant treatments.^8^ However, no recent studies have yet focused on the treatment indications of antidepressants and the potential associated factors in Danish older adults, despite the fact that the use of off‐label medications is widespread in Denmark, and older adults are more vulnerable to adverse‐drug‐reactions from the potential unapproved drug use.

We, therefore, aimed to describe the patterns and trends of treatment indications of antidepressants redeemed by older adults at community pharmacies in Denmark and explore factors associated with potential off‐label antidepressant use by considering prescriptions with missing and unspecified treatment indications.

## 2 METHODS

### 2.1 Study population, design and data materials

We included individuals aged ≥65 years living in Denmark anytime during the study period of 2006–2019 and followed a cross‐sectional study design. We describe the frequencies and trends of treatment indications of antidepressants by including all prescriptions with antidepressants redeemed by the older adults in 2006–2019 at Danish community pharmacies. To explore factors associated with a prescription of antidepressants with a specific indication, we only considered the first prescription with antidepressants, identified by Anatomical Therapeutic Chemical (ATC) code N06A. This study considered prevalent prescribing, meaning included individuals may have a record of using antidepressants before their inclusion. The Danish Civil Registration System (CRS) was established in 1968, where all persons alive and residing in Denmark are registered.^19^ CRS includes information on the date of birth, sex, marital status, and a unique personal identification number (CPR).^19^ Using CPR, we were able to link data from the Danish National Prescription Register (DNPR), which covers information on all prescriptions redeemed at community pharmacies from 1995 onwards in Denmark.^20^ DNPR contains information on dispensed drug names, the ATC Classification codes, indications, and dispensation dates.^20^

We obtained socio‐demographic information from the Population Statistics Register, and the Population's Education Register,^21^ and clinical factors (e.g., date and diagnoses of hospital contacts) from the Danish National Patient Register.^22^ Included categorical variables were age in years (65–70/71–75/76–80/≥81), sex (male/female), years of schooling as education (>12 as long/10 to 12 as medium/<10 as short), marital status (single/widow (er)/separated/married or registered partnership), place of residence (capital region/north Jutland/central Jutland/southern Denmark/Zealand), country of origin as ethnicity (Danish/western/non‐western/unknown), and calendar year of antidepressants prescription (2006–2012/2013–2014/2015–2019) with the first category as the reference. We also included continuous variables including within the last 10 years the number of past somatic diagnoses assessed as Charlson comorbidity Index score, number of past psychiatric diagnoses (based on one digit of ‘F’ codes 0–9 in ICD10), within last year number of somatic and psychiatric hospital contacts (separately), and number of different drug use (ATC 7digit level). All the variables were assessed at the first prescription.

### 2.2 Statistical analyses

We present frequencies and percentages of indications by antidepressant classes at the prescription level, meaning indication for each antidepressant prescription was counted once and was summed up as total count by antidepressant classes. The classes are (i). Selective Serotonin Reuptake Inhibitors (SSRIs): N06AB, (ii). Noradrenergic and Specific Serotonergic Antidepressants (NASSAs): N06AX03, N06AX11, (iii). Serotonin‐Norepinephrine Reuptake Inhibitors (SNRIs): N06AX16, N06AX21, (iv). Tricyclic antidepressants (TCAs): N06AA, and (v). Other antidepressants (Noradrenaline Reuptake Inhibitor, monoamine oxidase inhibitor [MAO‐I NS] and others): N06AX18, N06AF, N06AG, N06AX12, N06AX22, N06AX26.

To investigate whether the treatment indication for dispensed antidepressants changed over time, we calculated the proportion (presented as a percentage) of specific indications in 11 major categories, for example, depression, sedation (sedatives or tranquilizers referring Danish term nerve medicine), anxiety, pain, motion sickness, tobacco cessation, OCD, insomnia, others, unspecified and missing by calendar years from 2006 to 2019 for all issued prescriptions in that years for each drug class.

For exploring factors associated with off‐label antidepressant use, we only considered the first antidepressant prescription, and prescriptions with missing and unspecified indications were analyzed using a multinomial logistic regression analysis with ‘depression’ indication as the reference category and reported odds ratios (OR) with 95% confidence intervals. As TCAs users are likely to be different from the rest of the users, in a sensitivity analysis, we excluded individuals with TCAs at their first prescription and rerun the association analysis. In an additional analysis, we reran the same model, where we included three separate variables, including whether individuals' had dementia, depression, and other psychiatric diagnoses, instead of applying the past psychiatric diagnoses as a continuous variable to see whether the likelihood differs in those with depression and dementia diagnosis.

All data management and statistical analyses were performed in SAS version 9.2 (SAS Institute Inc).

## 3 RESULTS

**FIGURE 1:**
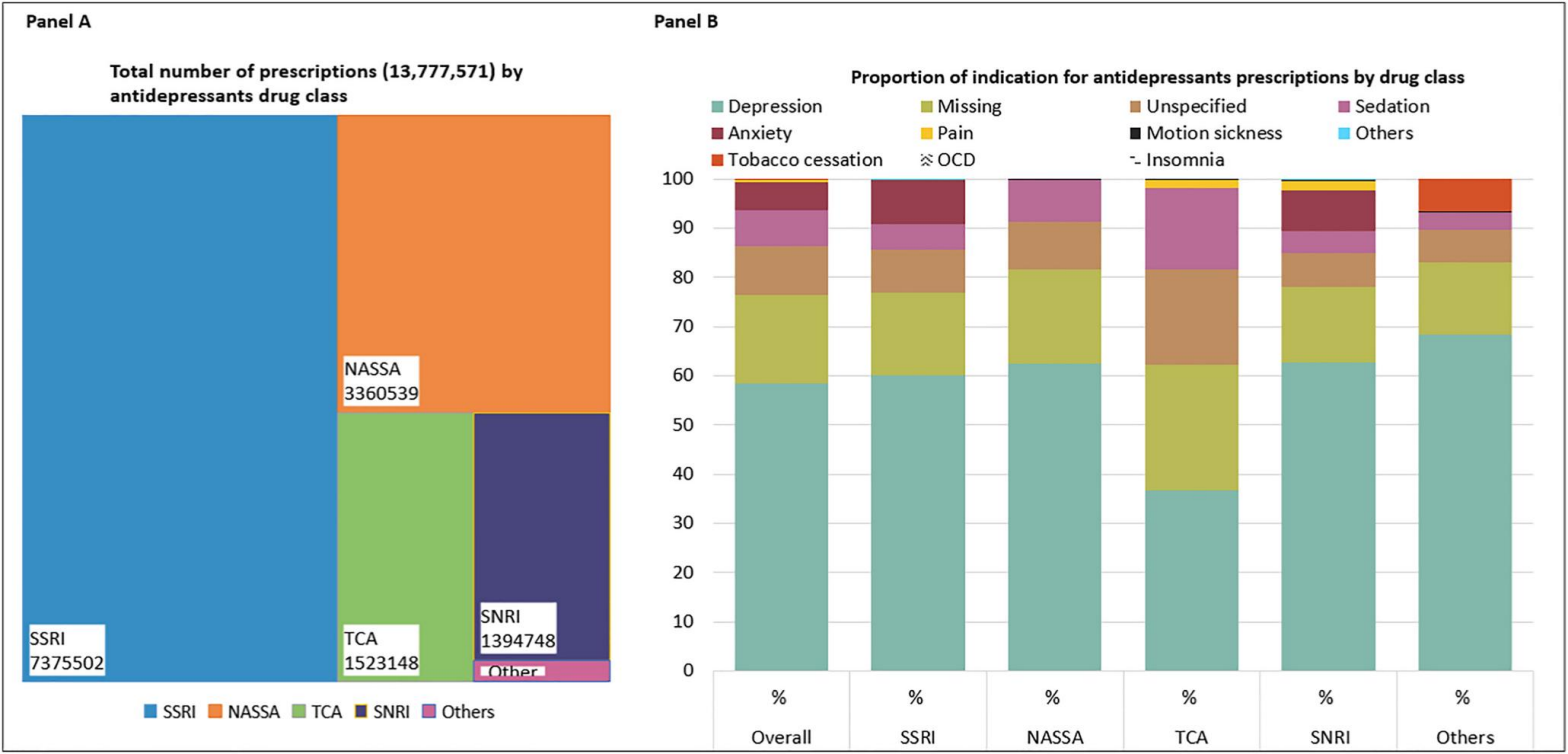
Proportion of treatment indication for antidepressant prescriptions redeemed by older adults at community pharmacies in Denmark from 2006 to 2019 [Number of older adults: 462,657; Total prescriptions: 13, 777, 571].

We identified 462,657 older adults who had redeemed a total of 13, 777, 571 prescriptions with antidepressants during 2006–2019 at the primary care pharmacies in Denmark. Overall, SSRIs (53.5%) were the most prescribed antidepressants, followed by NASSAs (24.4%), TCAs (11.1%), SNRIs (10.1%), and others (0.9%) (Figure 1, Panel A). Supplement Table S1 shows detailed characteristics of older adults redeeming their first antidepressant prescription at community pharmacies in Denmark in 2006–2018 (*N* = 215,352).

### 3.1 Frequencies and pattern of treatment indications and off‐label use

The most common indication for all antidepressants was depression (58.4%), followed by tranquillizers (7.2%), anxiety (5.7%), pain (0.4%), motion sickness (0.1%), tobacco cessation (0.1%), and others (0.1%) (Figure 1, Panel B; Supplement Table S2). Of all antidepressant prescriptions, 18.1% had missing indications, and 9.9% had unspecified indications, totaling 28% of prescriptions as potential off‐label use. According to drug class, depression was the most common indication (over 60%) in all drug classes, except for TCAs, where the proportion was nearly 37%. The indication was missing in prescriptions with TCAs 25.6%, NASSAs 19.1%, SSRIs 16.6%, SNRIs 15.4%, and others 14.6%, while indication was unspecified in TCAs 19.2%, NASSAs 9.7%, SSRI 8.7%, SNRIs 7.0%, others 6.8%.

### 3.2 Trend in patterns of treatment indications and off‐label use

**FIGURE 2:**
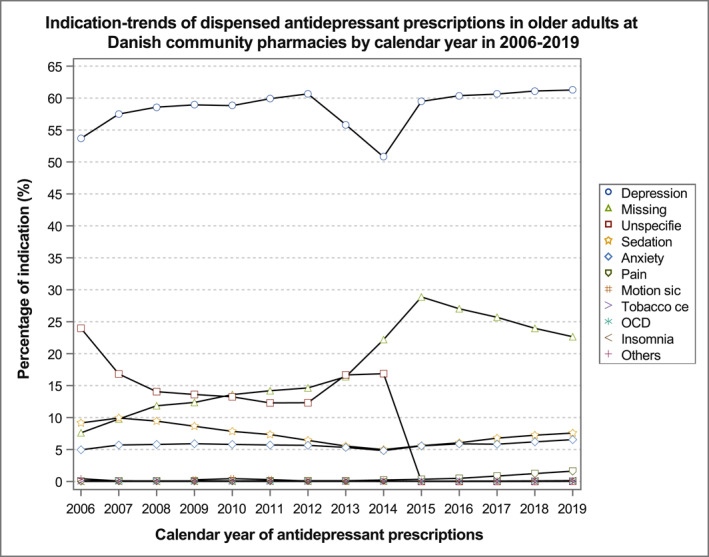
Trend in patterns of treatment indications of older adults redeemed prescriptions with antidepressants at community pharmacies in Denmark from 2006 to 2019.

The proportion of prescribed antidepressants for depression indication remained high consistently at around 60%, with a fluctuation in 2013 and 2014 at around 50% (Figure 2). The proportion of the missing indication gradually increased until 2013 and experienced a steep increase from 16% in 2013% to 29% in 2015, and then it gradually reduced and remained above 20%. In contrast, the proportion of the unspecified indication gradually decreased with a bump in 2013–2014, where it increased from 12% in 2012% to 17% in 2014 but sharply dropped to 0% in 2015–2019. Although the proportion of missing and unspecified prescriptions combined, potential off‐label use remained between 25% and 30%. The proportion of prescriptions with sedation indication constantly remained below 10%, and it gradually decreased over the years until 2014, then increased slightly from 2015 to 2019. A similar trend was observed for prescriptions with the indication of anxiety, although for most of the years, the proportion remained just below the proportion of sedation of 10%. For the remaining indications, the changes are not noticeable, and they closely overlapped each other with a proportion below 1%.

### 3.3 Factors associated with missing and unspecified indications for the first prescription with antidepressants

Characteristics associated with a higher likelihood of missing indication than depression were female sex (vs. male: OR 1.05, 95% CI: 1.02–1.07), and married/registered partnership (vs. single: 1.20, 1.12–1.28), and more recent years of prescription (e.g., in 2015–2019 vs. 2006–2012: 2.02, 95% CI: 1.96–2.07) (Figure 3A). A lower chance of missing indication for antidepressants was associated with increased age (≥81 vs. 65–70 years: 0.68, 95% CI: 0.66–0.71), shorter educational attainment (short vs. long: 0.90, 95% CI: 0.87–0.94), place of residence other than capital region (e.g., northern Jutland vs. capital region: 0.51, 0.49–0.54), and the number of past psychiatric diagnoses (per each: 0.76, 0.73–0.78).

FIGURE 3(A) Factors associated with off‐label use (prescriptions with missing and unspecified indications) for the first prescription with antidepressants in Danish older adults (Reference indication: Depression, *N* = 215352). (B) Sensitivity analysis of factors associated off‐label use (prescriptions with missing and unspecified indications) for the first prescription with antidepressants in Danish older adults after excluding prescriptions with TCAs (Reference indication: Depression, *N* = 180230).

**Figure:**
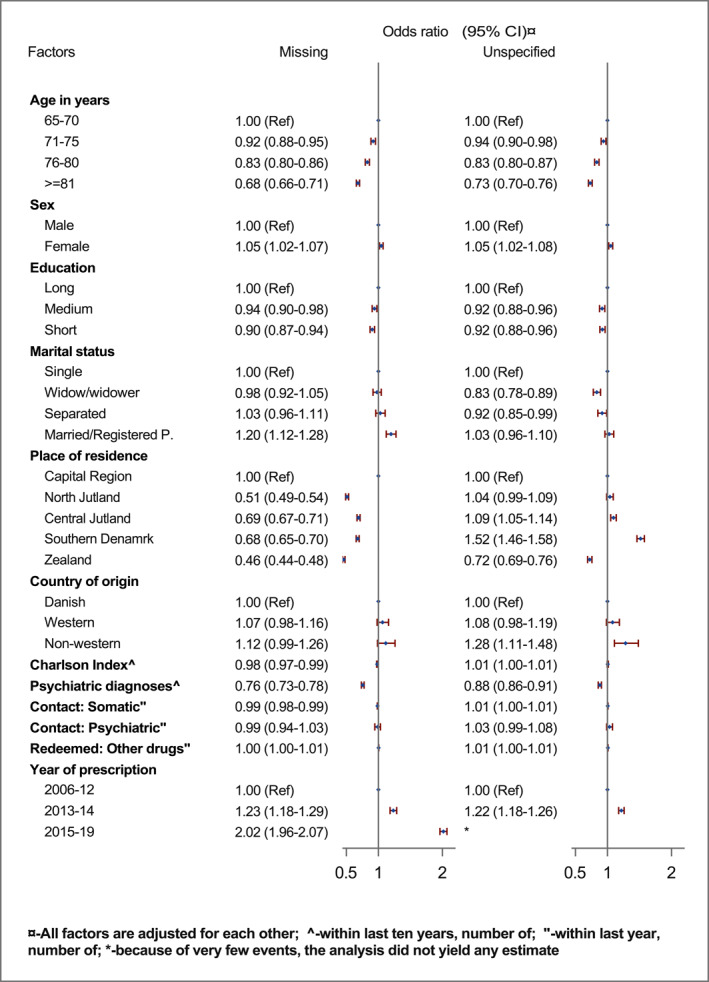

**Figure:**
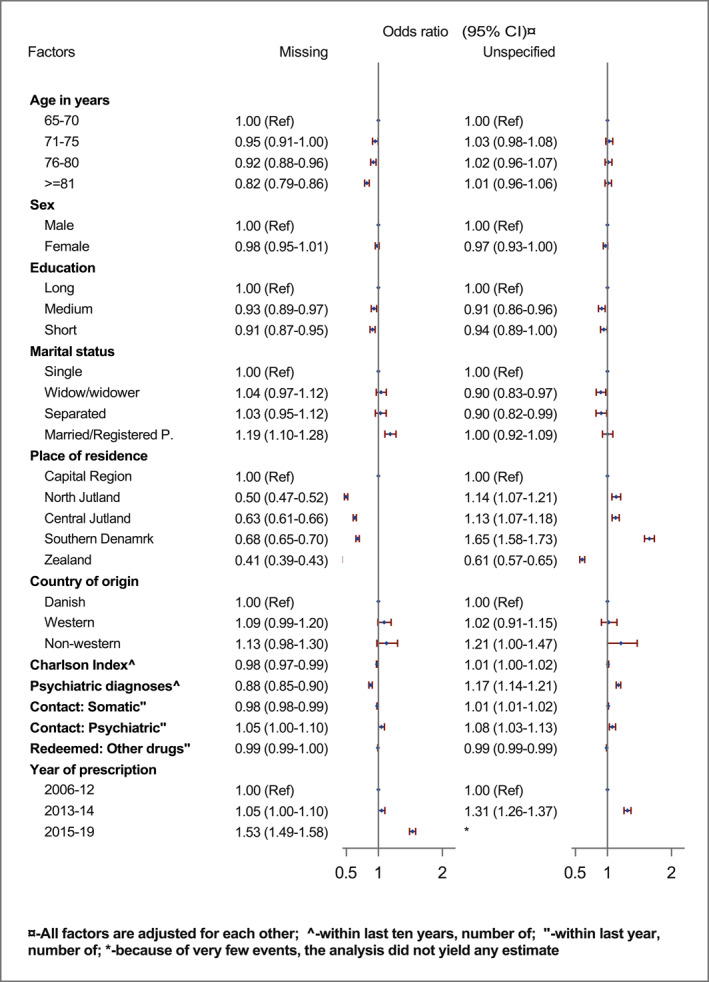

Similar factors and direction of association were also yielded for prescriptions with unspecified indications with only exceptions that a higher OR was associated with place of residence as either central Jutland or southern Denmark (e.g., southern Denmark vs. capital region 1.52, 1.46–1.58), and ethnicity as non‐western (vs. Danish 1.28, 1.11–148).

Results from the sensitivity analysis after excluding older adults with TCAs, in general, corroborated the results from the main analysis, except a higher OR of receiving a prescription with unspecified indication was associated with the number of past psychiatric diagnoses (1.18, 95% CI: 1.14–1.22) (Figure 3B). Moreover, age was no longer associated with unspecified indication, and sex was no longer associated with the missing and unspecified indication. Additional analysis indicated that past diagnoses of dementia and depression were associated with a lower likelihood of using prescriptions with missing and unspecified indications (Supplement Figure 1).

## 4 DISCUSSION

### 4.1 Main findings

In this descriptive study, we found depression was the most common indication for treatment, accounting for approximately 60% of all antidepressant prescriptions redeemed by older adults in Denmark during 2006–2019. Over 18% of all prescriptions had a missing indication, and 9.9% had unspecified indication meaning nearly one in three antidepressants prescriptions in Danish older adults may not have an approved treatment indication. Drug class‐wise, prescriptions with TCAs had the highest proportion of missing and unspecified indications, followed by NASAs, SSRIs, SNRIs, and others. Noticeable changes in the proportion of antidepressants appeared in 2013–2015, in which indication with depression dropped while missing indications increased. However, considering the prescriptions with missing and unspecified indications combined, the potential off‐label use seems to have remained mostly stable during the entire period. Being female, married, or in a registered partnership, non‐western and more recent years of prescription increased the odds of redeeming the first antidepressant prescriptions with either a missing or/and unspecified indication rather than depression. Increased age, shorter educational attainment, place of residence other than Capital region, and having past psychiatric diagnoses were associated with lower odds of redeeming antidepressant prescriptions with either missing or/and unspecified indication.

### 4.2 Comparison with previous literature and interpretation of the main findings

#### 4.2.1 Prevalence and trend of treatment indication for antidepressants and off‐label use

We are not aware of any Danish studies reporting indications of antidepressant prescriptions redeemed by older adults. Existing studies on antidepressants prescriptions in Denmark, in general, focused on drug utilization patterns. Although a few studies had exclusively focused on indication or off‐label use of antidepressants, they only included either pregnant women^23^ or populations aged <18 years^14^ or only included a single antidepressant (Mirtazapine).^24^ A recent Danish study focusing on indications of redeemed antidepressants by pregnant women in 2006–2016 reported a lower proportion of missing and unspecified indications (21% combined) than our study.^23^ Compared with pregnant women, older adults are likely to have diverse psychiatric/somatic conditions requiring treatment with antidepressants,^25^, ^26^ which may lead to a higher proportion of missing/unspecified indications.

A multinational study on indications of prescribed antidepressants in older adults in Canada, Taiwan, the UK, and the USA in 2009–2014 reported that chronic pain was the most common indication, followed by depression, anxiety, major mental illness.^27^ Potential differences in treatment guidelines and health care systems across the nations, use of proxy measures for indication such as using a record of health problems from medical services billing data or electronic medical record data, and exclusion of individuals with antidepressant prescriptions within 2 years before inclusion in the multinational study may explain the differences with our results. In line with our study, a Dutch study on dispensed antidepressants at the primary care pharmacies in 1996–2012 reported that depression was the most common indication for prescribing antidepressants followed by anxiety, sleep disorders, and neuropathic pain.^28^

About 28% of all antidepressant prescriptions in our study had either missing or unspecified indications, and drug‐class‐wise TCAs had the highest proportion of indications missing and unspecified. We do not know if they were actual off‐label uses. Antidepressants' off‐label use has been reported as 44% in German older adults in 2009–2015,^12^ 40% in Danish children and adolescents in 2006%–2012%, and 29% in a general population of Quebec, Canada in 2003–2015 where TCAs had the highest prevalence of off‐label use.^11^, ^12^ Differences in definition and assessment criteria (off‐label use in previous studies vs. missing indication in our study), year of study, and country‐specific treatment guidelines perhaps explain the discrepancies in the proportion of off‐label use across the studies.

Concerning indication trends, like our study, Liu and colleagues reported a drop in the proportion for depression in 2014–2016, yet, they only reported data until 2016.^23^ The proportion of prescriptions with unspecified indications was zero in 2015–2019, which possibly contributed to an average increase in the proportion of missing in 2015–2019 than in previous years. Though, the trend of missing indications gradually reduced in 2015–2019.

We speculate that the changes in the trends of depression, missing and unspecified indications in 2013–2015 could be a result of several changes in the guidelines related to antidepressant treatment and primary care pharmacies in Denmark in 2013–2015, for example, a new guideline for antidepressant treatment in 2015,^29^ and a nationwide reduction in drug prices at community pharmacies in 2013.^30^ More importantly, this might also be due to technical changes in data reporting in the DNPR—a new software system was introduced and replaced the existing one in 2011.^31^ However, implementation of the new system was completed by all sectors only in 2015. As part of the transition from the old to the new system (records transferring), there was an agreement regarding prescription documentation between the involved healthcare providers across sectors, including Danish municipalities, regions, and GPs, which expired in 2015.^32^ This change in the transition presumably contributed to the disappearance of unspecified indications and adding them to the missing indication category.

#### 4.2.2 Patient‐related factors and missing and unspecified indication for the first antidepressant prescription

Most of the studies on the indication of antidepressants did not include socio‐demographic and clinical information. Only a few included socio‐demographics but did not analyze them as a determinant. Previous studies reported that females tend to use antidepressants without reporting depression, whereas males are less likely to use antidepressants while having depression symptoms.^33^, ^34^ Moreover, females are more positive about seeking mental health care than males, suggesting higher use of treatment in females than males.^35^ Although we found being female was associated with higher odds of missing/unspecified indications, the point estimate was small and was reduced to null when TCAs were excluded from the analysis.

Individuals who are married or living with partners are more likely to seek health care and treatment than those who live alone or separated.^36^ This could be one of the explanations that in our data, married/living with partners had higher odds of receiving prescriptions with missing indications since they might be prone to use antidepressants for uncommon purposes, or they might have very mild symptoms to be labeled with depression or similar diagnosis. Like‐wise, this presumably also explains the lower odds of unspecified prescription use in individuals who were widows/widowers/separated.

Older age, short education, living in a city, and past psychiatric diagnoses are often considered risk factors for depression in older adults.^37^ They may increase the chance of receiving an antidepressant prescription for depression while reducing the possibility of missing/unspecified indications of antidepressant prescriptions.^37^ That probably explains our findings of lower odds of redeeming antidepressant prescriptions with a missing/unspecified indication in older adults with increased age, lower educational attainment, place of residence other than Capital region (concentration of major urban areas in Denmark), and having past psychiatric diagnoses. Moreover, it has been documented in a Danish study that patients with lower socioeconomic status and longer distance between residence and health care services are associated with lower use of mental health care regardless of free or paid services,^38^ suggesting perhaps a lower use of antidepressants in them. That also probably supports the idea that prescriptions with missing/unspecified indications are linked to over and under‐use antidepressant treatment. Regional differences in prescriptions with missing/unspecified indications may partly be explained by the potential differences in the software system transitions (from PEM to CMC) in 2011–2015.^31^

It is not surprising that the older adults with past psychiatric diagnoses were less likely to receive a prescription with missing/unspecified indication than for depression, suggesting those with missing/unspecified indications of antidepressants might have less severe depression/psychiatric conditions.

As we included prevalent prescription users, the cases in the early years are likely to be prevalent cases, whereas the cases in the later years as more incident cases. Thus, the observed calendar year effect concerning missing/unspecified indications is conceivably to reflect the prevalent versus incident case differences rather than an actual effect of the calendar year.

### 4.3 Strength and limitations

The main strength of our study lies in its register‐based design, with a large representative study population. The DNPR has widely been used in scientific research as a sole source of information on dispensed prescriptions in the primary care pharmacies in Denmark and has been shown to be highly accurate.^20^ We included wide‐ranging socio‐demographical and clinical information from highly accurate national registers, and missing information was negligible.

The study has several limitations. First, the DNPR does not include information on diagnoses linking to each prescription. The use of the indication variable in the DNPR for identifying treatment indications has not been validated yet in an empirical study. This limitation might have affected the validity of our study greatly. However, several previous studies used the variable and values to determine treatment indications.^23^, ^39^ Based on the current data, we cannot confirm whether prescriptions with missing or unspecified indications were actually off‐label use as we do not know the reasoning behind the missing indications and the actual indication of those prescriptions with unspecified indications. One explanation for prescriptions with missing indications could be that prescribers, particularly GPs, are often under work pressure in Denmark (e.g., handling a higher number of patients per week compared with GPs in other Nordic countries,^40^ also experience work‐related burnout^41^), leading to incomplete information for a prescription. Association analyses suggested similar factors with the same direction of the association, indicating the differences between missing and unspecified indications are perhaps irrelevant to users but are a matter of documentation technicality. Second, the prescriptions with documented indications (e.g., sedation, OCD, pain) may not necessarily indicate that they were on‐label, which we did not explore. Consequently, defining off‐label use based on missing and unspecified treatment indications might have led to an underestimation of the actual off‐label use Third, we did not assess off‐label use by dosage, another key criterion often used in defining and characterizing off‐label use. Over 62% of the prescriptions do not have dosage information. Besides, we do not have information on individuals' weight, the severity of the condition, and pharmacokinetic considerations, which are essential besides age and sex for determining whether the prescribed dosage was off‐label. Like‐wise, we did not include prescription duration, which could also be meaningful in defining off‐label use. These aspects should be focused on future research besides treatment indications to illustrate a detailed picture of off‐label use. Fourth, we do not have access to information about prescribers. There may be a difference in the treatment indications used by GPs/private psychiatrists and by the prescribers at the hospitals. A Danish study assessed that up to 90% of all depression treatments initiated at non‐hospital based settings. This unmeasured factor is to some extent reflected in past hospital contacts, which we included in the analyses. Therefore, prescriber differences might not have a substantial impact on our results. Moreover, we included previous somatic and psychiatric hospital contacts and diagnoses, which may have accounted for the chances of one being prescribed antidepressants either from a hospital or outside. Fifth, we do not know about patients' self‐awareness of mental health and their role in the communication with the doctor and the treatment decision‐making process. We included information on educational and marital status. These may have captured some differences in patient‐doctor communication and health literacy. Finally, the study results may only be applied to Denmark or other Nordic countries, since the guidelines for treating with antidepressants and the health care system, in general, differ from country to country.

In conclusion, nearly one‐third of all antidepressant prescriptions redeemed by older adults in Danish primary care pharmacies in 2006–2019 had either missing or unspecified indications inferring potential off‐label use. As expected, the most common indication was depression. Overall, the trends of potential off‐label use (missing and unspecified indications combined) remained mostly stable during the study period, with a few notable changes in 2013–2015. Several patient‐related factors, including age, sex, education, place of residence, and past psychiatric diagnosis, were associated with potential off‐label antidepressant use. Future studies should investigate the potential consequences of using antidepressants with missing and unspecified indications on the clinical outcomes. The study findings might be of clinicians' interest for understanding the current uses of treatment indications and paying attention to patients' characteristics in prescribing antidepressants with missing and unspecified indication in older adults. Moreover, we emphasize the importance of maintaining good documentation while prescribing medicine to this vulnerable group to promote safe and easier medication reviews.

## AUTHOR CONTRIBUTIONS

**Kazi Ishtiak‐Ahmed**: Conceptualization; Data curation; Formal analysis; Funding acquisition; Investigation; Methodology; Project administration; Resources; Software; Visualization; writing – original draft; Writing – review & editing. **Xiaoqin Liu**: Conceptualization; Methodology; Writing – review & editing. **Kaj Sparle Christensen**: Conceptualization; Writing – review & editing. **Christiane Gasse**: Conceptualization; Resources; Methodology; Supervision; Writing – review & editing.

## CONFLICT OF INTEREST

The authors declare no conflict of interest.

## ETHICS STATEMENT

The study was approved by the Danish Data Protection Agency, and no further ethical approval is required regarding register‐based research in Denmark.

## Supporting information

Supporting Information S1

figure S1

## Data Availability

The data used in this study are from Statistics Denmark. They are stored in an anonymous form and are not publicly available. To use such data for research, require special permission in compliance with the Danish Data Privacy Act.
