# Supplementary material for: Treatment indications and potential off‐label use of antidepressants among older adults: A population‐based descriptive study in Denmark
Source: Int J Geriatr Psychiatry. 2022 Nov 15;37(12):10.1002/gps.5841. doi: 10.1002/gps.5841 (PMC9828742; doi:10.1002/gps.5841)
Supplement: Supplementary file 1 — Supporting Information S1 [file GPS-37-0-s002.docx]

**Supplement table 1:** Characteristics of older adults who redeemed the first antidepressant prescriptions at the community pharmacies in Denmark during 2006-2018 (from the total of 215,352 individuals, only 208,524 individuals are shown covering the five most frequent treatment indications of antidepressant prescriptions)

|  | Depression, N=110,209 | Potential off-label use | | Sedation, N=18,711 | Anxiety, N=11,425 |
| --- | --- | --- | --- | --- | --- |
|  |  | Missing, N=36,089 | Unspecified, N=32,090 |  |  |
|  | (%) | (%) | (%) | (%) | (%) |
| Age in years |  |  |  |  |  |
| Age65-70 | 17.8 | 21.6 | 23.2 | 20.2 | 24.6 |
| Age71-75 | 19.9 | 23.2 | 22.4 | 22.5 | 23.9 |
| Age76-80 | 21.2 | 21.8 | 21.3 | 21.6 | 20.0 |
| Age81-85 | 41.2 | 33.4 | 33.1 | 35.8 | 31.5 |
| Sex |  |  |  |  |  |
| Male | 44.8 | 45.2 | 45.2 | 46.2 | 36.1 |
| Female | 55.2 | 54.8 | 54.8 | 53.8 | 63.9 |
| Education |  |  |  |  |  |
| Short | 49.0 | 44.6 | 50.4 | 48.5 | 48.0 |
| Medium | 32.9 | 36.2 | 31.5 | 34.2 | 34.7 |
| Long | 11.6 | 14.7 | 11.5 | 11.8 | 11.9 |
| Missing | 6.5 | 4.5 | 6.5 | 5.5 | 5.4 |
| Marital status |  |  |  |  |  |
| Single | 4.6 | 4.4 | 4.6 | 4.8 | 5.1 |
| Widow/widoer | 38.1 | 31.3 | 33.3 | 31.8 | 32.6 |
| Separated | 11.2 | 11.6 | 10.8 | 11.2 | 12.8 |
| Married/registered p. | 45.9 | 52.6 | 51.1 | 52.2 | 49.4 |
| Missing | 0.2 | 0.2 | 0.2 | 0.1 | 0.2 |
| Place of residence |  |  |  |  |  |
| Capital region | 24.3 | 33.9 | 23.0 | 22.8 | 28.7 |
| North Jutland | 12.1 | 9.0 | 11.2 | 10.5 | 10.9 |
| Central Jutland | 23.5 | 23.6 | 23.4 | 25.4 | 21.4 |
| Southern Denmark | 23.5 | 22.7 | 31.1 | 27.8 | 21.4 |
| Zealand | 16.5 | 10.7 | 11.1 | 13.4 | 17.3 |
| Missing | 0.2 | 0.2 | 0.2 | 0.1 | 0.2 |
| Country of origin |  |  |  |  |  |
| Danish | 96.1 | 95.1 | 95.7 | 95.3 | 95.3 |
| Western | 2.4 | 2.8 | 2.5 | 2.7 | 2.9 |
| Non-western | 1.5 | 2.1 | 1.8 | 1.9 | 1.8 |
| Within last ten years: |  |  |  |  |  |
| Had a dementia diagnosis |  |  |  |  |  |
| No | 87.3 | 91.2 | 89.6 | 89.9 | 92.9 |
| Yes | 12.7 | 8.8 | 10.4 | 10.1 | 7.1 |
| Had a depression diagnosis |  |  |  |  |  |
| No | 94.3 | 97.1 | 95.2 | 98.4 | 98.3 |
| Yes | 5.7 | 2.9 | 4.8 | 1.6 | 1.7 |
| Year (inclusion) |  |  |  |  |  |
| 2006-2012 | 57.1 | 43.1 | 80.0 | 48.8 | 50.7 |
| 2013-2014 | 11.7 | 10.7 | 20.0 * | 9.4 | 11.6 |
| 2015-2019 | 31.2 | 46.2 |  | 41.8 | 37.8 |
|  | Mean (standard deviation) | Mean (standard deviation) | Mean (standard deviation) | Mean (standard deviation) | Mean (standard deviation) |
| Within last ten years: |  |  |  |  |  |
| Charlson index score (based on somatic diagnoses) | 1.63 (1.84) | 1.52 (1.82) | 1.66 (1.88) | 1.51 (1.83) | 1.24 (1.64) |
| Number of psychiatric diagnoses | 0.28 (0.58) | 0.19 (0.49) | 0.24 (0.55) | 0.20 (0.50) | 0.20 (0.49) |
| Within last year: |  |  |  |  |  |
| Number of somatic contacts | 3.53 (4.10) | 3.29 (3.99) | 3.87 (4.33) | 3.25 (3.97) | 2.95 (3.77) |
| Number of psychiatric contact | 0.08 (0.37) | 0.05 (0.30) | 0.07 (0.33) | 0.05 (0.34) | 0.06 (0.33) |
| Number of other drug use | 9.69 (5.70) | 9.43 (5.50) | 9.77 (5.71) | 9.80 (5.63) | 9.14 (5.63) |

*For unspecified indication in the year category '2015-2019' had observation smaller than five, which is merged with the previous cell

Supplement Table 2: Frequencies of indication for antidepressant prescriptions redeemed by older adults at community pharmacies in Denmark from 2006 to 2019 [Number of older adults: 462,657; Total prescriptions: 13,777,571]

| Treatment indication | Overall | | SSRI | | NASSA | | TCA | | SNRI | | Others | |
| --- | --- | --- | --- | --- | --- | --- | --- | --- | --- | --- | --- | --- |
|  | N | (%) | N | (%) | N | (%) | N | (%) | N | (%) | N | (%) |
| Depression | 8,048,633 | (58.4) | 4,437,273 | (60.2) | 2,095,332 | (62.4) | 559,088 | (36.7) | 872,531 | (62.6) | 84,409 | (68.3) |
| Missing* | 2,491,640 | (18.1) | 1,226,448 | (16.6) | 641,870 | (19.1) | 389,937 | (25.6) | 215,390 | (15.4) | 17,995 | (14.6) |
| Unspecified* | 1,364,985 | (9.9) | 641,424 | (8.7) | 324,812 | (9.7) | 292,667 | (19.2) | 97,725 | (7.0) | 8,357 | (6.8) |
| Sedation | 996,278 | (7.2) | 387,590 | (5.3) | 288,415 | (8.6) | 252,541 | (16.6) | 63,382 | (4.5) | 4,350 | (3.5) |
| Anxiety | 783,578 | (5.7) | 665,422 | (9.0) | 2,472 | (0.1) | 1,031 | (0.1) | 114,648 | (8.2) | 5 | (0.0) |
| Pain | 52,159 | (0.4) | 213 | (0.0) | 319 | (0.0) | 25,578 | (1.7) | 26,049 | (1.9) | 0 | (0.0) |
| Motion sickness | 11,244 | (0.1) | 6,141 | (0.1) | 2,582 | (0.1) | 886 | (0.1) | 1453 | (0.1) | 182 | (0.1) |
| Others | 10,729 | (0.1) | 4,942 | (0.1) | 1,076 | (0.0) | 1,109 | (0.1) | 3,570 | (0.3) | 32 | (0.0) |
| Tobacco cessation | 8,293 | (0.1) | 0 | 0 | 0 | 0 | 0 | 0 | 0 | 0 | 8,293 | (6.7) |
| OCD | 6,030 | (0.0) | 6,030 | (0.1) | 0 | 0 | 0 | (0.0) | 0 | 0 | 0 | 0 |
| Insomnia | 4,002 | (0.0) | 19 | (0.0) | 3,661 | (0.1) | 311 | (0.0) | 0 | 0 | 11 | (0.0) |

OCD: Obsessive-compulsive disorder, SSRIs: Selective Serotonin Reuptake Inhibitors, NASSAs: Noradrenergic and Specific Serotonergic Antidepressants, SNRIs: Serotonin-Norepinephrine Reuptake Inhibitors, TCAs: Tricyclic antidepressants, Other: Noradrenaline Reuptake Inhibitor, monoamine oxidase inhibitor [MAO-I NS] and others;

#For Pain indication, less than five in other drug group, so we merged with SNRI; for OCD indication, less than five in TCA drug group, so we merged with SSRI

* Potential off-label use
