# Supplementary material for: Treatment indications and potential off‐label use of antidepressants among older adults: A population‐based descriptive study in Denmark
Source: Int J Geriatr Psychiatry. 2022 Nov 15;37(12):10.1002/gps.5841. doi: 10.1002/gps.5841 (PMC9828742; doi:10.1002/gps.5841)
Supplement: Supplementary file 2 — figure S1 [file GPS-37-0-s001.pdf]

Figure S1: Additional analysis of factors associated with off-label use (prescriptions with missing and unspecified indications) for the first prescription with antidepressants in Danish older adults (Reference indication: Depression, N=215352)

Factors	Odds ratio (95% CI)¤ Missing	Unspecified
**Age in years**		
**65-70**	1.00 (Ref)	1.00 (Ref)
**71-75**	0.93 (0.89-0.96)	0.94 (0.90-0.98)
**76-80**	0.84 (0.81-0.87)	0.84 (0.80-0.87)
**>=81**	0.70 (0.67-0.72)	0.74 (0.71-0.77)
**Sex**		
Male	1.00 (Ref)	1.00 (Ref)
Female	1.05 (1.02-1.08)	1.05 (1.02-1.08)
**Education**		
Long	1.00 (Ref)	1.00 (Ref)
Medium	0.94 (0.90-0.98)	0.92 (0.88-0.96)
Short	0.90 (0.87-0.94)	0.92 (0.88-0.96)
**Marital status**		
Single	1.00 (Ref)	1.00 (Ref)
Widow/widower	1.00 (0.93-1.06)	0.84 (0.78-0.90)
Separated	1.04 (0.97-1.12)	0.92 (0.86-1.00)
Married/Register P.	1.22 (1.15-1.30)	1.03 (0.97-1.10)
**Place of residence**		
Capital Region	1.00 (Ref)	1.00 (Ref)
North Jutland	0.51 (0.49-0.54)	1.04 (0.99-1.09)
Central Jutland	0.70 (0.67-0.72)	1.10 (1.05-1.14)
Southern Denamrk	0.68 (0.65-0.70)	1.51 (1.46-1.57)
Zealand	0.46 (0.44-0.48)	0.73 (0.69-0.76)
**Country of origin**		
Danish	1.00 (Ref)	1.00 (Ref)
Western	1.06 (0.98-1.16)	1.08 (0.98-1.19)
Non-western	1.12 (0.99-1.26)	1.28 (1.11-1.48)
**Had a dementia diag.^**		
No	1.00 (Ref)	1.00 (Ref)
Yes	0.75 (0.71-0.78)	0.89 (0.85-0.93)
**Had a depression diag.^**		
No	1.00 (Ref)	1.00 (Ref)
Yes	0.58 (0.53-0.62)	0.79 (0.74-0.84)
**Had other psyc. diag.^**		
No	1.00 (Ref)	1.00 (Ref)
Yes	0.86 (0.82-0.91)	0.86 (0.81-0.91)
**Charlson Index^**	0.98 (0.97-0.99)	1.01 (1.00-1.01)
**Contact: Somatic"**	0.99 (0.98-0.99)	1.01 (1.00-1.01)
**Contact: Psychiatric"**	0.95 (0.91-1.00)	1.02 (0.98-1.07)
**Redeemed: Other drugs"**	1.00 (1.00-1.01)	1.01 (1.00-1.01)
**Year**		
2006-12	1.00 (Ref)	1.00 (Ref)
2013-14	1.23 (1.18-1.28)	1.22 (1.17-1.26)
2015-19	2.00 (1.95-2.05)	*

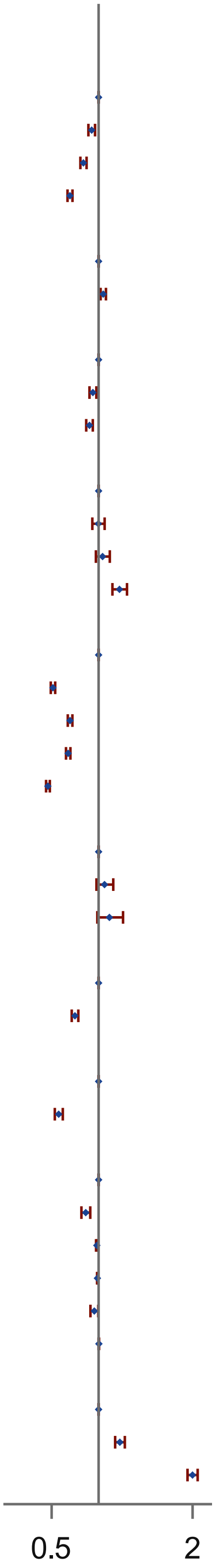

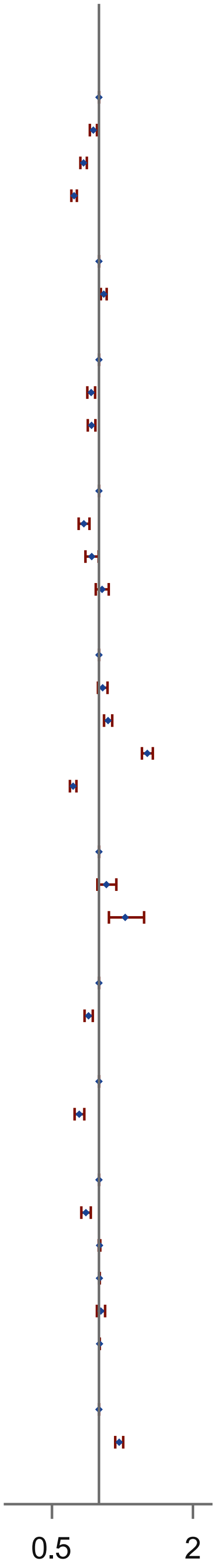

¤-All factors are adjusted for each other; ^-within last ten years, number of; "-within last year, number of; diag.-diagnosis; psyc.-psychiatric; *-because of very few events, the analysis did not yield any estimate
